# When Pedestrian Crossings Become Danger Zones: Trauma and Mortality Risks in Elderly Pedestrians

**DOI:** 10.3390/ijerph22101556

**Published:** 2025-10-13

**Authors:** Peter Pavol, Vasileios Topalis, Sofia-Chrysovalantou Zagalioti, Olha Kuzyo, Martin Müller, Aristomenis K. Exadaktylos, Mairi Ziaka, Jolanta Klukowska-Rötzler

**Affiliations:** 1 Department of Emergency Medicine, Inselspital University Hospital Bern, University of Bern, 3010 Bern, Switzerlandvtopalismd@gmail.com (V.T.); olha.kuzyo@insel.ch (O.K.); martin.mueller@insel.ch (M.M.); mairi.ziaka@gmail.com (M.Z.); 2 Department of Emergency Medicine, AHEPA University General Hospital, Aristotle University of Thessaloniki, 54636 Thessaloniki, Greece; 3 Academic Department of Emergency Medicine, School of Medicine, University of Cyprus, 1678 Nicosia, Cyprus; aexadaktylos@hin.ch; 4 University of Bern, 3010 Bern, Switzerland

**Keywords:** aging population, crosswalk accidents, elderly, pedestrians, zebra crossings

## Abstract

Aim: Older adult pedestrians are at greater risk of severe injuries than younger pedestrians due to gradual physical changes and coexisting medical conditions. This leads to longer hospital stays, increased mortality risk, and higher inpatient costs. Focusing on the aging population, this study explores the characteristics and injury profiles of pedestrian crossing accidents in the capital city of Bern, Switzerland. Methods: Our retrospective cohort study comprised adult patients admitted to our ED between 1 January 2013 and 31 December 2023, as crossing (or zebra crossing)-related pedestrian victims. Two cohorts were formed on the basis of age < 65 and ≥65 years and compared according to the setting of the accident, type, pattern of the injury, and clinical outcomes (short-term mortality, ICU/hospital length of stay). Results: Of a total of 124 patients, 31.5% (n = 39) of patients were elderly (65+ group). In contrast to the younger patients, the aging population was predominantly admitted as inpatients (64.1% vs. 35.3%, *p* = 0.001) and was hospitalised in the intensive care unit (20.5% vs. 6%, *p* = 0.020). Older patients were more likely to be polytraumatised (41% vs. 11.8%, *p* = 0.001) and to have been tossed or hurled than patients under 65 years (75% vs. 47.3%, *p* = 0.016). Fractures of the upper extremities (17.9% vs. 4.7%, *p* = 0.016), pelvis (30.8% vs. 9.4%, *p* = 0.003), and thoracic spine (12.8% vs. 2.4%, *p* = 0.019) were significantly more common in the elderly population. Intracranial haemorrhage (35.9% vs. 17.6%, *p* = 0.026), abdominal trauma (17.9% vs. 5.9%, *p* = 0.035), and relevant vessel damage (30.8% vs. 3.5%, *p* < 0.001) were also significantly higher in geriatric patients. Trauma indices were slightly more increased in the older population than in the younger group (ISS; *p* = 0.004 and AIS > 2 of chest and thoracic spine; abdomen, pelvic contents, and lumbar spine; extremities & bony pelvis *p* < 0.05). The 65+ group had a longer length of hospital stay (*p* = 0.001) and ICU stay (*p* = 0.002). A hospital stay longer than 7 days was also significantly more common in elderly individuals (*p* = 0.007). In-hospital (15.4% vs. 1.2%, *p* = 0.001) and 30-day mortality (17.9% vs. 1.2%, *p* < 0.001) were significantly higher in patients over 65 years of age. Conclusion: In our study, the impact of pedestrian crossing accidents was more severe in the elderly, as indicated by the severity of injuries, hospitalisation rate, longer length of hospital and ICU stays, and higher mortality rates. These findings underline the importance of developing tailored strategies to reduce crosswalk accidents and to optimise management approaches for these vulnerable patients.

## 1. Introduction

One in four older adults falls each year, making falls the leading cause of injury among adults aged 65 and older, and resulting in significant morbidity and mortality [1,2,3]. In addition, life expectancy is expected to rise by 2025, with the world’s geriatric population growing from 600 million in 2000 to 1.2 billion by 2025 [4], and this highlights the demographic shift that contributes to the rising burden of fall-related injuries, pedestrian accidents, and associated healthcare costs [5].

Switzerland has made substantial progress in road safety over the past two decades, achieving a 60% reduction in road fatalities between 2000 and 2023 [6]. Nevertheless, 236 people were killed in road accidents in 2023, with pedestrians representing a significant share [6]. Despite high-quality infrastructure and strict traffic regulations, older pedestrians remain particularly vulnerable. This concern is highlighted by demographic trends: in 2023, 19.61% of the Swiss population—approximately 1.73 million individuals—were aged 65 or older, reflecting a steady rise from previous years [7]. At the European level, the share of the population aged 65 and over is projected to reach 30% by 2060, making population aging a major public health consideration across the continent [8].

With the global population aging at an unprecedented rate, road safety for elderly pedestrians has emerged as a critical public health challenge. According to the WHO, pedestrian fatalities accounted for 19% of all road traffic deaths worldwide in 2020, with nearly half of these victims aged 65 and older [9,10,11,12,13]. Elderly pedestrians are particularly vulnerable, due to age-related physiological changes, including slower reflexes, impaired vision, and underlying health conditions [12], which is important, as fall-related injuries are more severe with age [14]. Compared to inactive older adults, who experience most falls indoors, active older adults primarily fall outdoors [15,16]. Epidemiological research has concluded that the percentage of patients who had severe injuries from falls that happened indoors and outdoors (on streets or pavements) are either similar, or that indoor falls were related to less-severe injuries [17,18]. The latter finding may be because home-related falls frequently happening on a single level [18]. Furthermore, falls that occur at home typically lack any additional risks, such as high speed or collision, which could result in more injuries [18].

According to WHO data, falls in Switzerland in 2023 are one of the top causes of death [19]. Additionally, physical activity levels have increased remarkably in Switzerland [20] and this may explain the possible rise in falls in public spaces. In general, walking or mild activity is favourable for the elderly, as it can reduce cardiovascular risk and enhance muscle strength and balance [21,22]. In accordance with these benefits, older adults are adopting a more active lifestyle; so, levels of activity are clearly increasing over time [23]. However, as more people choose to walk, the likelihood of pedestrian accidents has grown, and age is a significant risk factor [24,25]. In 2020, pedestrian fatalities were 19% of all road traffic deaths worldwide, and 49% were related to patients aged over 65 [13]. Older adult pedestrians are at greater risk of severe injuries than younger pedestrians [26].

While previous large-scale studies, such as that of Siram et al. (2011) [27], have described injury patterns and outcomes among elderly pedestrians using U.S. trauma registry data, there remains a lack of detailed, context-specific analyses focusing on crosswalk-related accidents in high-income European settings with advanced pedestrian infrastructure. To date, limited literature exists regarding environmental contributors to elderly pedestrian collisions. For instance, a study conducted in Madrid highlighted the role of infrastructure design in improving safety for older adults [28].

Furthermore, Oxley et al. (1997) demonstrated age-related differences in traffic judgment, indicating that older pedestrians often underestimate traffic risks, which contributes to risky crossing behaviors [29]. Dommes et al. (2013) found that functional declines in vision and mobility predict hazardous crossing decisions among elderly pedestrians [30]. A review by Tournier et al. (2016) comprehensively synthetized international evidence on elderly pedestrian safety, emphasizing the importance of urban design and behavioral interventions to reduce risks [31].

Moreover, an analysis of hospital discharge data from eight European countries showed that head, spinal and lower extremities injuries are the most frequent and life-threatening outcomes in pedestrian collisions. However, the disproportionately high mortality rates among elderly pedestrians remain insufficiently explained [32]. Further evidence from Finland demonstrated that elderly pedestrians face significantly higher fatality rates, particularly at pedestrian crossings, even in settings with well-developed pedestrian infrastructure [33].

Walking represents a key element of transport systems, exhibiting diverse patterns across countries, cities, and even within different areas of the same city. These patterns are influenced by factors such as trip length, gender, age, and income, which affect both walking behavior and pedestrian exposure. Despite these variations, a major concern consistently emerges across settings: pedestrian safety, particularly among vulnerable age groups, including the elderly [34]. Indeed, population-related injury rates for pedestrians older than 85 years in Switzerland exceed 25 per 100,000 inhabitants [35]. Nevertheless, despite the well-developed pedestrian infrastructure in high-income countries such as Switzerland, the disproportionately high mortality rates among elderly pedestrians have not been adequately explained. This concern motivated the present study, which focuses on zebra-crossing accidents aiming to bridge the existing knowledge gap by analyzing crossing-related pedestrian accidents in Bern, Switzerland, over the past ten years, with a particular focus on injury patterns, trauma severity, and patient outcomes. Specifically, this research seeks to compare the nature and severity of injuries, as well as mortality outcomes, and comparing elderly pedestrians (≥65 years) and their younger counterparts (<65 years). Furthermore, we aim to evaluate the association between trauma severity indices, including the Injury Severity Score (ISS) and the Abbreviated Injury Scale (AIS), and key patient outcomes, such as hospital length of stay, intensive care unit (ICU) admissions, and in-hospital mortality. In addition, this study investigates potential modifiable risk factors contributing to severe injuries in elderly pedestrians, and offers insights into urban traffic safety and emergency care strategies to mitigate the burden of pedestrian injuries in an aging population. By providing analyzing modifiable risk factors unique to the Swiss context, this research offers new insights for targeted prevention and management strategies tailored to aging urban populations, relevant to countries with advanced pedestrian infrastructure, in order to inform both clinical practice and public policy.

## 2. Materials and Methods

### 2.1. Data Collection and Retrospective Survey

This research was carried out at a Level I trauma center located in Bern, Switzerland (University Emergency Centre, Inselspital). This institution manages over 55,406 cases annually (data from 2024) and the analysis focused on demographic and health-related information of patients older than 16 years. Patients younger than 16 were excluded, as they are admitted to a dedicated pediatric emergency department.

This study focused exclusively on pedestrian accidents occurring at marked crossings (zebra crossings). These incidents were the only ones reliably identifiable in our electronic hospital records using specific keywords such as “Fussgänger” (pedestrian) and “Zebra” (crossing). Other types of pedestrian accidents, such as those occurring mid-block or at unmarked intersections, were not consistently documented in a searchable format and were therefore excluded. This selection also allowed for a more homogeneous study population, reducing variability in accident setting and enabling clearer comparisons between age groups.

Patients who sustained acute trauma at designated pedestrian crossings between 1 January 2013, and 31 December 2023, were identified by searching our computerized patient database E.care (E.care BVBA, ED 2.1.3.0, Turnhout, Belgium) using the keywords “Fussgänger” (pedestrian) and “Zebra” (crossing) in either the diagnosis or medical history fields. Records of duplicate cases and outpatient follow-up visits were excluded from the analysis. In addition, cases involving referees or coaches, injuries related to overuse, or those with insufficient information were omitted. Patients who had not provided general consent, or who later withdrew their consent for the use of their data, were also excluded from the study cohort.

Each consultation was analysed to obtain detailed information on the reason for the medical consultation. The primary objective was to compare two age groups: individuals aged 65 years and older (≥65 years) and those younger than 65 years (<65 years). The analysis aimed to identify significant differences in demographics, consultation characteristics, accident types, injury patterns, trauma severity, and outcomes in patients older than 65 years compared to younger individuals.

### 2.2. Study Cohort and Data Sources

This study encompassed all patients who presented to a Level 1 trauma centre following a road traffic accident and as pedestrians at marked crossings from 2013 to 2023. Patient data were extracted from electronic medical records, which included detailed demographic information, clinical presentations, and specific information on the incidents. Search terms utilised for data retrieval comprised “Fussgänger” (pedestrian) and “Zebra” (crosswalk or crossing). All data were identified according to institutional privacy protection standards before analysis. A unique study identifier replaced all original patient identifiers to enable data linkage while maintaining anonymity.

We assessed re-identification risk using established criteria for small datasets. No individual record contained a unique combination of quasi-identifiers that would enable singling out. Data suppression was applied to cells containing fewer than 3 cases to prevent potential identification through rare characteristic combinations. The dataset was reviewed by our institutional privacy board before analysis to ensure adequate protection levels.

### 2.3. Demographics

Demographic data were systematically categorised to facilitate a precise analysis. Participants were stratified into two age groups: individuals under 65 years of age and those aged 65 years and older. The age cutoff of ≥65 years was chosen based on established geriatric trauma literature, which identifies this threshold as clinically significant due to increased morbidity and mortality, age-related physiological vulnerability, and its widespread use in standardized trauma triage protocols [36,37,38]. Gender was documented as male or female. This demographic categorisation permitted clear comparisons between the two age groups and their respective characteristics.

### 2.4. Consultation Characteristics

Details of patient consultations were meticulously recorded, and encompassed various aspects of patient presentations. Data were collected for the year and month of the presentation, covering each year from 2013 to 2023 and each month from January to December. The day of the week and time of presentation were also noted, with time categorised into four intervals: 0:01–6:00, 6:01–12:00, 12:01–18:00, and 18:01–0:00. Furthermore, the mode of presentation to the hospital was documented, including self-admission, ambulance transport, air ambulance transport, external hospital transfer, and referrals from family physicians or emergency medical services.

In addition, details of the patient’s status after consultation were recorded. Patients were classified by whether they were discharged from the hospital, returned home, admitted to the hospital, died in the emergency department, transferred to another facility, hospitalised in a specific department, or returned home after transfer. Moreover, data were documented regarding the departments to which patients were admitted, including general internal medicine, neurology and neurosurgery, intensive care, orthopaedics, hand surgery, thoracic surgery, visceral surgery, stroke units, and rheumatology. This detailed characterisation of consultations aimed to enhance the understanding of patient needs and the course of their treatment.

### 2.5. Accident Type

The type of accident was classified based on the vehicles involved. Accidents were categorized by the type of vehicle, including cars, bicycles, e-bicycles, e-scooters, trams, motorcycles, buses, or trucks. The time of the incident was noted to indicate whether it occurred during the day or at night. The reported vehicle speeds were typically derived from patient statements, eyewitness accounts, or, in some cases, information conveyed by emergency medical services. Assessments of vehicle speed focused on whether the pedestrian was struck or hurled, with the impact side classified as lateral, frontal, or rear. This comprehensive classification of accident types aimed to provide a clear understanding of the circumstances surrounding the incidents.

### 2.6. Injury Characteristics

Details regarding injuries were collected to gain insights into the nature and severity of injuries sustained by pedestrians. Types of injuries were documented as monotrauma, polytrauma, multiple injuries, or cases in which the individual was unconscious after the accident. The Glasgow Coma Scale (GCS) score was recorded upon hospital admission. “Polytrauma” refers to severe, life-threatening injuries involving multiple body regions, requiring coordinated critical care. “Multiple injuries,” in contrast, indicate the presence of multiple injuries that may not be life-threatening and do not necessarily require intensive intervention [26]. Specific types of fractures were categorised, including head fractures, cervical spine fractures, thoracic spine fractures, and fractures of the upper and lower extremities. Additionally, other regions of trauma were documented, including traumatic brain injury (TBI), intracranial haemorrhage (ICH), and abdominal trauma with associated vascular injuries.

In this study, TBI was defined as any injury to the brain parenchyma, skull, or intracranial space, and severity was assessed using the GCS upon admission, regardless of imaging findings. ICH was analyzed separately and included subdural, epidural, subarachnoid, or intraparenchymal haemorrhage, as identified by computed tomography (CT) imaging. While ICH represents a subtype of TBI, it was reported distinctly due to its clinical relevance and frequency in the elderly subgroup. Thus, differences in ICH prevalence do not necessarily correspond with differences in overall TBI severity as measured by GCS.

### 2.7. Trauma Severity

The severity of injuries was assessed using established scoring systems. The ISS was calculated based on recorded injuries. The AIS was also employed to evaluate the severity of injuries across various anatomical regions, including head/neck, face, chest, abdomen, spine, pelvis, and extremities. This thorough assessment of injury severity provided a comprehensive overview of the injuries sustained by pedestrians.

### 2.8. Outcomes

Outcomes were evaluated as based on the length of hospital stay, with patients classified according to length of stay (LOS ≥ 7 days and LOS < 7 days). The number of surgical procedures performed and the duration of stay in the ICU were also recorded. Furthermore, in-hospital mortality and 30-day mortality rates were documented to assess the overall outcomes associated with pedestrian accidents at marked crossings.

### 2.9. Statistical Analyses

Statistical analyses were performed using STATA version 18.1 (StataCorp, College Station, TX, USA). Continuous variables were reported either as means with standard deviations (SD) or as medians with interquartile ranges (IQR), depending on the outcome of the Shapiro–Wilk normality test. Comparisons between two independent groups (e.g., males vs. females) were assessed using the unpaired *t* test for normally distributed data and the Wilcoxon rank-sum test for non-normally distributed data. Categorical variables were summarized as frequencies and percentages, and group differences were evaluated using the chi-square test.

For categorical variables, *p*-values were obtained using chi-square tests. In instances where expected frequencies were <5 (e.g., in some yearly or time-of-day subgroup analyses), Fisher’s exact test was additionally applied to confirm the results, with no discrepancies observed.

To explore independent predictors of 30-day mortality, we performed multivariable logistic regression analyses. Variables entered into the models included geriatric status (≥65 years vs. <65 years), mechanism of injury (two-axis vehicle involvement), and region-specific severe injuries (AIS ≥ 3 for head, face, chest, or abdomen). Separate models were constructed for each injury region to avoid overfitting and to account for the limited number of outcome events. Odds ratios (ORs) with 95% confidence intervals (CIs) were calculated.

### 2.10. Statistical Tests and Rationale

The choice of statistical tests was guided by both the scale of measurement and the underlying distributional properties of the data. The Shapiro–Wilk test was applied to continuous variables to formally evaluate the assumption of normality. This test is widely regarded as one of the most powerful procedures for detecting departures from normality, particularly in small to moderate samples, and is therefore well suited to our dataset. Establishing whether the normality assumption held was essential in order to determine the appropriate statistical framework and to avoid inflated type I or type II error rates.

For variables that met the assumption of normal distribution, data were expressed as mean ± standard deviation and compared between groups using the independent samples *t*-test, which provides optimal power under parametric conditions. For variables that did not follow a normal distribution according to the Shapiro–Wilk test, data were summarized as median with interquartile range and compared using the Wilcoxon rank-sum test, a non-parametric alternative that does not rely on distributional assumptions and is robust to outliers.

Categorical variables were summarized as frequencies and percentages. Differences between groups were tested using Pearson’s chi-square test, which is appropriate when expected cell counts are sufficiently large. When expected frequencies fell below five, Fisher’s exact test was used instead, as it provides an exact *p*-value and avoids approximation errors in small samples (Table S1).

Finally, in order to explore independent predictors of 30-day mortality, we employed multivariable logistic regression analyses. Logistic regression is the standard approach for binary outcomes, and in our study design, it allowed for adjustment of clinically relevant covariates. Because of the limited sample size and event counts, these analyses were interpreted as exploratory and hypothesis-generating, with full acknowledgement of their limitations.

By tailoring each statistical test to the type and distribution of the data, our analytical approach maximized statistical validity and minimized the risk of spurious conclusions arising from inappropriate test selection. A schematic overview of the statistical analyses is presented in Flowchart 1B.

## 3. Results

### 3.1. Design

**Figure 1:**
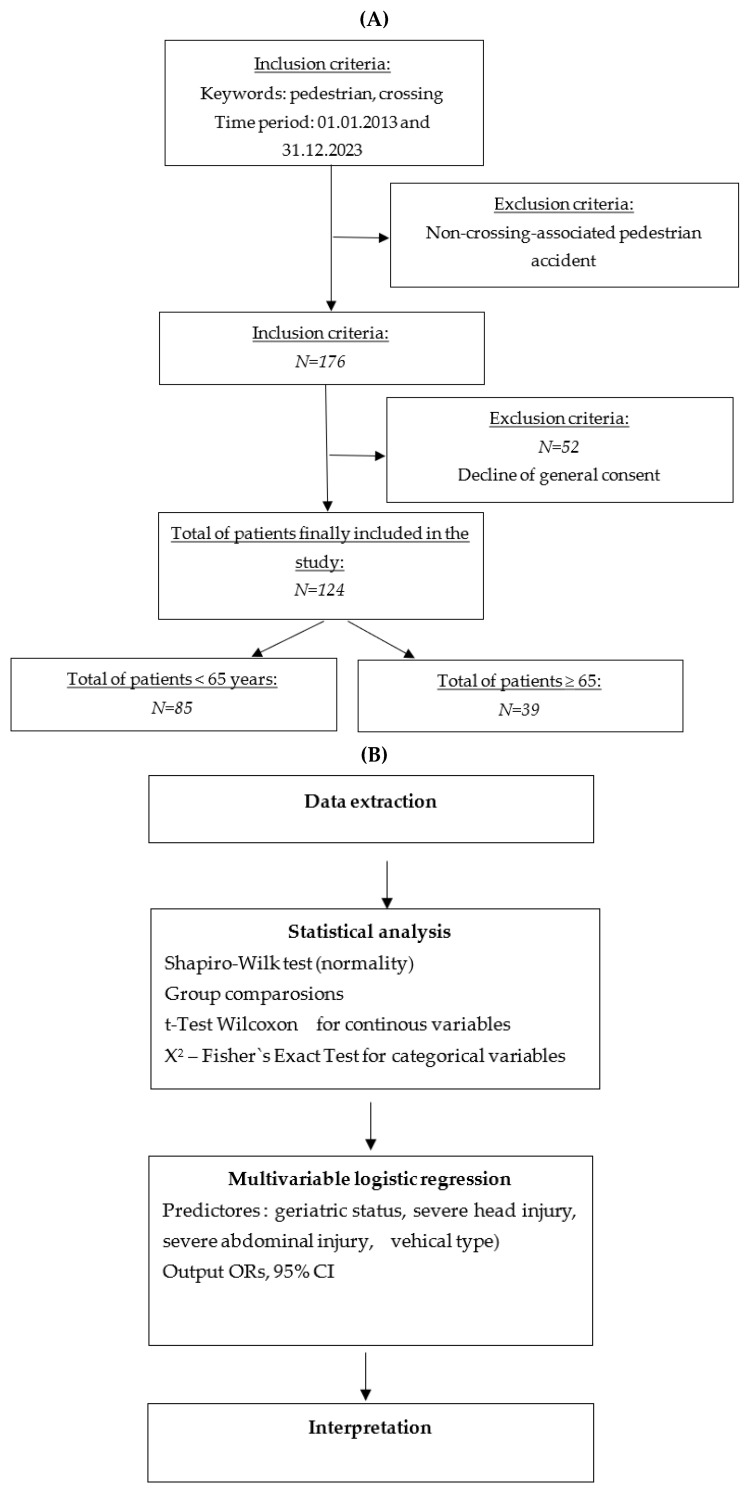
(**A**) Flow chart illustrating the exclusion and inclusion procedure in this study. (**B**) Overview of statistical methods applied in this study.

Between 1 January 2013, and 31 December 2023, a total of 628 patients were identified as having been involved in pedestrian accidents occurring on marked crossings. Excluded from this study were 452 patients whose admission was not linked to the pedestrian accident, along with an additional 52 patients due to lack of concordance with the general consent. Ultimately, 124 patients who met all criteria were included in this study (Figure 1).

### 3.2. Demographics

Over the study period, a total of 124 patients were admitted to our ED due to crossing-related pedestrian injuries (85 patients under 65 years of age and 39 elderly patients; *p* < 0.001). The median age was 39 years (26–54) for younger patients and 78 years (73–84) for elderly patients. Females constituted the majority in both age groups, representing 62.4% of younger and 56.4% of elderly patients (*p* = 0.530). 57.6% of patients in age group < 65 years (n = 49) and 92.3% of patients in age group ≥ 65 years (n = 36) were Swiss (*p* < 0.001).

**Figure 2:**
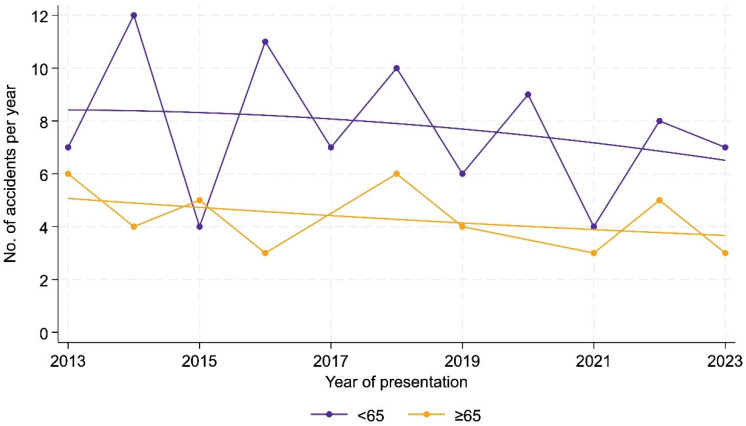
Annual distribution of younger (<65 years) and elderly (≥65 years) patients between 2013 and 2023. *p*-values were calculated using chi-square tests; Fisher’s exact test was additionally applied where expected frequencies were <5, yielding consistent results.

Over the study period, younger patients (<65 years) tended to consistently account for higher admission rates than elderly patients (≥65 years)—nearly half of the study period. Notable differences occurred in 2017 and 2020, where no elderly patients were admitted, while younger patients represented 8.2% and 10.6% of study population aged <65 years, respectively. In 2013 and 2018, elderly patients exceeded younger admissions (15.4% of the older study population each year), as well as in 2015 and 2022 (12.8% each year) (all *p* = 0.204) (Figure 2).

Although the difference did not reach statistical significance (*p* = 0.120), a seasonal pattern was observed. Younger patients (<65 years) had higher admission rates than elderly patients (≥65 years) during several months, particularly in November (14.1% vs. 7.7%), October (11.8% vs. 10.3%) and February (11.8% vs. 0%). Conversely, elderly admissions peaked in December (28.2% vs. 8.2%) and were also elevated in January (17.9% vs. 14.1%). These trends suggest a potential winter concentration of incidents among older individuals, which warrants further investigation in larger samples. For weekday patterns, younger patients had higher admission rates on Fridays (20.0%) and Mondays (17.6%), while elderly patients had the highest rates on Mondays (25.6%) and Wednesdays (23.1%) (*p* = 0.245). No significant seasonal or weekly pattern was evident overall. Most pedestrian accidents (43.5%) took place from 18.01–0.00 for the aged group under 65 years, in contrast with the aged group over 65 years (48.7%), where they took place from 12.01–18.00 (*p* = 0.014). In both patient groups, the fewest pedestrian accidents (4.8%) occurred from 0.01–6.00 (*p* = 0.014).

The mode of arrival differed between younger and older individuals: a greater proportion of younger patients were walk-ins (n = 15; 17.6%), compared to older patients (n = 2; 5.1%). Conversely, air-ambulance transfer rates and transfer rates from other hospitals were less common among younger patients (7.1% vs. 17.9% and 5.9% vs. 17.9%, respectively; *p* = 0.029). No repatriations from another country were reported for the older group (≥65 years), in contrast to 2.4% (n = 2) for the younger group.

In relation to the Swiss Emergency Triage Scale [39,40,41], we found no highly significant relationship between the two groups and their triage categories. 44 younger (51.8%) and 24 older adults (61.5%) were treated in our ED resuscitation room (*p* = 0.310). The characteristics of the two groups are presented in Table 1.

### 3.3. Discharge

Most patients in the younger group were discharged home, with 54 individuals (63.5%) compared to 11 older individuals (28.2%). Among the younger patients, 30 (35.3%) were admitted as inpatients, while 25 (64.1%) of the older patients were admitted (*p* = 0.001). In our ED, one patient from the younger group (1.2%) and one patient from the older group (2.6%) died (*p* = 0.001) (Table 1).

Hospitalised individuals were primarily admitted to four departments, which showed significant differences between the groups (*p* = 0.020). Specifically, 20.5% of older patients (n = 8) were hospitalised in the ICU, compared to 6% (n = 5) in the younger group. Additionally, 20.5% (n = 8) of the older patients were admitted to the orthopaedics department versus 14.3% (n = 8) among younger individuals. Furthermore, 15.4% (n = 6) of the elderly patients and 8.3% (n = 7) of younger patients were managed in the neurology/neurosurgery department. Finally, 10.3% (n = 4) of the older group and 1.2% (n = 1) of the younger group were admitted to the internal medicine department (Table 1).

### 3.4. Setting of the Accident

We analysed pedestrian-vehicle crash data to identify differences in the primary environments or settings for pedestrian accidents among age groups. Cars were by far the most common cause of accidents, accounting for 88.2% of incidents involving younger individuals and 87.2% involving older individuals. In the oldest group, the second most common cause was E-bikes (5.1%; n = 2), while for the youngest patients, it was bicycling (3.5%; n = 3) and motorcycles (3.5%; n = 3) (all *p* = 0.607). In both older (92.3%; n = 36) and younger patients (83.5%; n = 71), more accidents occurred during the day than at nighttime (all *p* = 0.187). The median vehicle speed was 30 km/h (15–40) for younger patients and 37.5 km/h (15–40) for patients in the age group over 65 years (*p* = 0.697). The proportion of patients who had been hurled was significantly higher in older patients (75%; n = 21) than in younger individuals (47.3%; n = 26; *p* = 0.016). Lateral crashes (91.1%; n = 112) were more common in both groups; the frontal impact was second most common (7.3%; n = 9), and rear crashes were only reported in elderly patients (5.3%; n = 2) (*p* = 0.100). The description of the accident setting for the two groups is presented in Table 2.

### 3.5. Type of Injury

Monotrauma was observed in 40% of younger individuals and 33.3% of older individuals, while the incidence of geriatric trauma was primarily associated with polytrauma (41%; n = 16 vs. 11.8%, n = 10) compared to younger individuals, where multiple injuries were more common (48.2%; n = 41 vs. 25.6%; n = 10) (*p* = 0.001). It is estimated that 31.8% (n = 27) of the group aged under 65 years experienced an episode of loss of consciousness after the accident, while the incidence in the group aged over 65 years was 28.2% (n = 11) (*p* = 0.690). The median score on the GCS was 15 for both groups; however, the IQR was 15–15 for younger patients and 13–15 for older patients (*p* = 0.008).

### 3.6. Pattern of Injury

**Figure 3:**
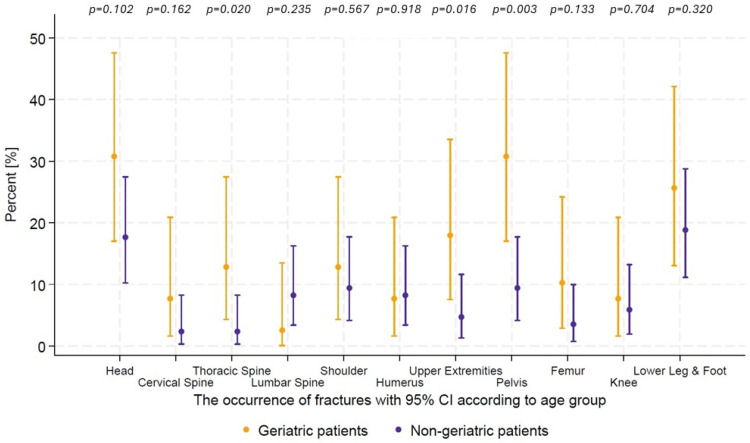
The occurrence of fractures according to age group.

The most common fracture sites for older adults were the head (30.8%; n = 12 vs. 17.6%; n = 15; *p* = 0.100) and the pelvis (30.8%; n = 12 vs. 9.4%; n = 8; *p* = 0.003). For younger adults, the most common sites included the head, the lower leg, and foot (17.6%; n = 15). Fractures of the thoracic spine were significantly more prevalent among older individuals (12.8%; n = 5 vs. 2.4%; n = 2, *p* = 0.019). Similarly, the incidence of upper extremity fractures was significantly higher in patients older than 65 years (17.9%; n = 7 vs. 4.7%; n = 4, *p* = 0.016). Although the difference did not reach statistical significance, fractures of the cervical spine (7.7%; n = 3 vs. 2.4%; n = 2) and femur fractures (10.3%; n = 4 vs. 3.5%; n = 3) were also more common among older patients (Figure 3).

**Figure 4:**
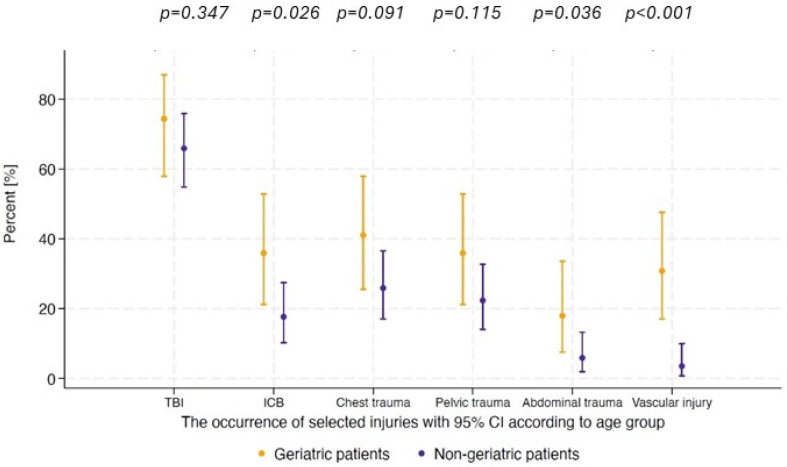
The occurrence of selected injuries according to age group.

The severity of TBI did not reach statistical significance between the groups. However, the rates of elderly patients diagnosed with ICH (35.9%; n = 14 vs. 17.6%; n = 15; *p* = 0.026), abdominal trauma (17.9%; n = 7 vs. 5.9%; n = 5, *p* = 0.035), and relevant vessel damage (30.8%; n = 12 vs. 3.5%; n = 3, *p* < 0.001) were significantly higher than those of patients aged under 65 years. Additionally, chest trauma, including contusio cordis (41%; n = 16 vs. 25.9%; n = 22), and pelvic trauma (35.9%; n = 14 vs. 22.4%; n = 19) were more common in the elderly population, although these differences were not statistically significant (Figure 4).

**Figure 5:**
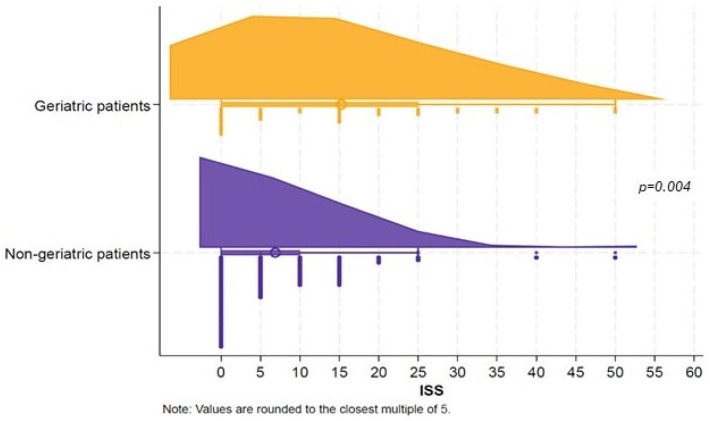
ISS distribution according to age group.

As an assessment of trauma severity, the ISS was significantly higher in adults aged 65+ (median 14; IQR 2–26) compared to younger patients (median 4; IQR 1–12; *p* = 0.004) (Figure 5).

**Figure 6:**
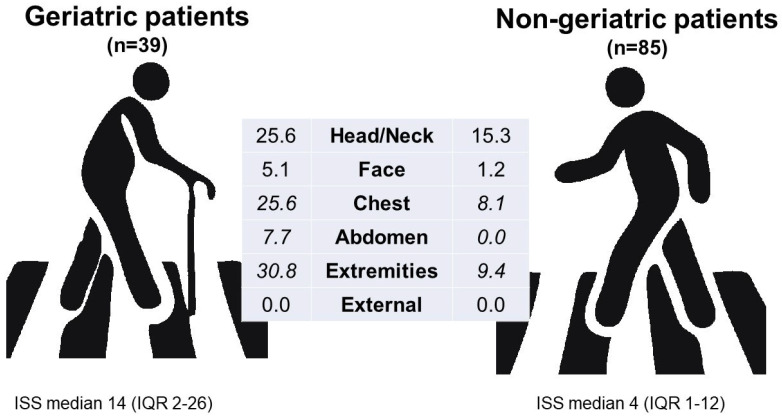
Severe body region trauma (AIS ≥ 3) in different AIS groups according to age group. Body regions with significant differences between the age groups are shown in italic.

Moreover, for the AIS score, chest injuries (25.6% vs. 8.2%, *p* = 0.009), abdominal injuries (7.7% vs. 0%, *p* = 0.010), and injuries to the extremities and pelvis (30.8% vs. 9.4%, *p* = 0.003) were significantly more severe in elderly patients (AIS score > 2) (Figure 6). Although head and neck injuries were more severe (AIS score > 2) in the older group (25.6% vs. 15.3%, *p* = 0.169), as were face injuries (5.1% vs. 1.2%, *p* = 0.184), these differences did not reach statistical significance. Overall, the injury patterns between the age groups are shown in Table 3.

### 3.7. Outcomes

A hospital stay of less than seven days was observed in 77.6% (n = 66) of younger patients and 53.8% (n = 21) of older individuals. Conversely, 46.2% (n = 18) of the geriatric group and 22.4% (n = 19) of patients under 65 had a hospital stay of seven days or longer (*p* = 0.007). Moreover, the duration of ICU hospitalisation was significantly higher among older individuals (*p* = 0.002).

In-hospital mortality was significantly higher for patients over 65 years (15.4%; n = 6 vs. 1.2%; n = 1; *p* = 0.001). Similarly, 30-day mortality differed significantly between groups (17.9%; n = 7 for older patients vs. 1.2%; n = 1 for younger adults; *p* < 0.001).

### 3.8. Multivariable Logistic Regression

In multivariable logistic regression analyses, geriatric status consistently emerged as a strong independent predictor of 30-day mortality across all models. Depending on the injury pattern included, geriatric patients had between a 9- and 13-fold higher risk of death compared with younger patients (OR range 9.26–13.14; all *p* < 0.05).

Among injury variables, severe head injury was independently associated with a markedly increased risk of 30-day mortality (OR 6.95–7.16, *p* = 0.01). Severe abdominal trauma was also significantly associated with mortality (OR 10.30, 95% CI 1.09–97.28, *p* = 0.042). In contrast, severe chest trauma showed only a statistical trend toward higher odds of death (OR 4.27, 95% CI 0.95–19.20, *p* = 0.059), and severe facial trauma was not significantly related to outcome (OR 0.59, 95% CI 0.02–17.21, *p* = 0.762).

Across all models, involvement of two-axis vehicles (i.e., powered two-wheelers in pedestrian accidents) was not significantly associated with 30-day mortality (ORs ranging 0.44–0.97, all *p* > 0.4).

## 4. Discussion

This study examines settings for pedestrian crossing accidents and related injuries in the aging population in a country where the quality of roads, particularly pedestrian crossings, is among the best worldwide [42]. As the population ages, there is growing concern for the safety of the elderly on the roads [43]. This is especially crucial for the elderly, as they are more vulnerable to accidents with more severe injuries [43]. Indeed, a comprehensive systematic review and meta-analysis by Azami-Aghdash et al. (2018) examining elderly pedestrian injuries across multiple high-income countries found that road traffic injuries comprised 23.6% of total injuries among elderly populations, with pedestrian injuries accounting for 48.1% of road traffic injuries [44]. This closely mirrors our study population demographics and confirms that elderly pedestrians represent a consistently vulnerable group across developed nations.

According to our results, patients aged 65+ were more likely to be polytraumatised patients, mainly with fractures in the pelvis and the thoracic spine. Additionally, we found that elderly patients suffer from ICH, abdominal trauma, and pelvic trauma with statistically significant frequency. Moreover, trauma indices were slightly more increased in the geriatric population than in younger patients. Elderly patients had a statistically significant higher ISS and a higher risk of serious injuries (AIS > 2) of the chest, abdomen, and extremities than patients < 65 years of age. According to previous research, pelvic injuries are associated with early death, and thoracic spine injuries have the potential to be life-threatening [45,46], suggesting that trauma to these body regions results in severe injuries for the patient. Notably, our results are consistent with those of a large retrospective analysis by Siram et al. (2011), which examined 79,307 pedestrian injuries and highlighted a significantly higher incidence of fractures and intracranial injuries among geriatric patients [27].

While severe TBI (defined as GCS ≤ 8) did not differ significantly between elderly and younger patients, the prevalence of ICH was significantly higher in the elderly group. This finding suggests that although the overall severity of TBI based on GCS was similar, elderly patients were more prone to haemorrhagic complications following trauma. ICH rates in our elderly population (35.9% vs. 17.6%) are consistent with findings from trauma registries in the United States, Canada, and Australia, where elderly patients consistently demonstrated higher rates of TBI complications. This pattern appears independent of the quality of emergency medical services or trauma care systems, suggesting an inherent age-related vulnerability to cerebrovascular complications following trauma [47,48,49,50].

The observed dissociation between TBI severity and ICH prevalence may be explained by age-related physiological differences. The elevated risk of intracranial bleeding among elderly individuals is likely multifactorial, involving age-related cerebral atrophy, increased vascular fragility, and the frequent use of antiplatelet or anticoagulant medications. These factors, often compounded by preexisting conditions such as arterial hypertension and specific trauma mechanisms, increase the likelihood of haemorrhage even in the absence of severe TBI as measured by the GCS. This highlights the need for elevated diagnostic vigilance in this vulnerable population [50,51,52,53,54].

As mentioned above, injury severity patterns observed in our study are also consistent with international research. McElroy and co-authors (2013) reported a median ISS of 12–13 for elderly patients, similar to our median ISS of 14 (IQR 2–26). However, elderly patients in both studies required ICU admission more frequently despite similar ISSs (61.6% vs. 40.2% in the Milwaukee study; 20.5% vs. 6% in our study) [55]. This pattern has been consistently reported across high-income countries, suggesting that age-related physiological vulnerability transcends healthcare system differences. The polytrauma rates in our elderly cohort (41% vs. 11.8% in younger patients) are higher than those reported in some international studies. Rod et al. (2021), in their systematic review, found that while injury severity was consistently higher in elderly pedestrians across studies, the specific proportion of polytrauma patients varied considerably between countries, likely reflecting differences in case definitions and trauma center criteria [56]. However, the overall trend of increased severe injury rates in elderly pedestrians was universal across all examined high-income countries.

Hospital LOS patterns from our study also mirror international trends. The study by McElroy et al. (2013) found no significant difference in overall hospital length of stay between elderly and non-elderly patients, but elderly patients were seven times more likely to be discharged to skilled nursing facilities [55]. Similarly, our study found significantly longer ICU stays and overall hospital stays for elderly patients, reflecting the complex recovery trajectory typical of geriatric trauma patients in developed countries. The higher percentage of ICU admissions and a longer LOS among geriatric patients compared to patients under 65 in our study may be associated with the significantly higher incidence of polytrauma, as well as higher ISS and AIS scores observed in the elderly population of our study. Our findings align with those of a systematic review and meta-analysis, which reported that injured pedestrians aged 60 and older, compared to those under 60, had a higher relative risk of severe injury, higher incidence rates of pedestrian falls leading to greater injury severity, more frequent ICU admissions, and higher fatality rates [56]. This is consistent with previous research showing that older age, polytrauma (compared to multiple injuries), and higher ISS are risk factors associated with more frequent inpatient treatment and prolonged LOS [57,58]. Similarly, Olszewski et al. (2015) identified the same risk factors for older people in a study conducted in Poland, which has Europe’s second-worst pedestrian fatality rate [59]. However, unlike in Switzerland, 30% of pedestrian injury accidents in Poland occurred at unsignalled zebra crosswalks [59]. Given that prolonged LOS is independently associated with a higher risk of complications [60], along with the greater severity of injuries in the 65+ group and the increased risk of death following ICU discharge, elderly pedestrian injury patients should be considered critically ill [61].

In our study, older individuals had significantly higher in-hospital and 30-day mortality, as is consistent with findings from previous studies [27,62]. Indeed, Azami-Aghdash et al. (2018) demonstrated a significant difference in mortality between elderly and non-elderly pedestrians (Odds Ratio = 2.57, 95% CI: 1.2–5.4) [44], which aligns with our findings of 17.9% versus 1.2% 30-day mortality rates. Similarly, a large single-center study conducted by McElroy et al. (2013) examining 945 pedestrian trauma patients found that elderly patients (≥65 years) had a mortality rate of 20.9% compared to 9.1% in non-elderly patients (*p* < 0.001) despite similar ISSs [55]. These findings support our observation that elderly patients experience disproportionately poor outcomes even with comparable trauma severity. A possible reason is that pedestrian-related injuries involve additional hazards, such as speed, collision, and victim ejection, all of which correlate with the severity of the injuries and contribute to the increased mortality rate among elderly patients [18]. Moreover, in addition to the higher severity of injuries in pedestrian accidents among older individuals, pre-existing comorbidities, associated medications such as oral anticoagulants, and a higher incidence of head injuries may contribute to the elevated mortality observed in this patient population [53,63,64,65]. In elderly polytraumatised patients, high trauma scores (ISS and AIS) are associated with an increased risk of death compared to younger patients [66,67]. This can be attributed to age-related changes in the physical and mental abilities of elderly pedestrians when crossing roads [68,69,70], which may explain our study’s findings. In our study, elderly individuals were hurled in a higher percentage than those under 65, probably due to instability, muscle loss (sarcopenia), and age-related decline in strength, all of which may contribute to the ejection of the victim [9,71].

In the multivariable logistic regression analyses, geriatric status consistently remained a strong independent predictor of 30-day mortality, and severe head and abdominal injuries also showed significant associations. In contrast, chest injuries demonstrated only a statistical trend, while facial injuries were not related to the risk of death. These findings are consistent with prior large-scale trauma registry studies, in which age and injury severity were identified as key determinants of survival [27,44]. Our regression analyses are subject to important limitations related to the small sample size and limited number of outcome events. Under such conditions, there is a high risk of unstable estimates, wide confidence intervals, and model overfitting. Even the use of penalized or exact logistic regression cannot completely resolve this issue. Consequently, the reported coefficients should be regarded as hypothesis-generating only, rather than definitive evidence. In clinical research in particular, the risk of drawing false conclusions from small datasets is well recognized and calls for considerable caution in interpretation.

The above-mentioned international comparisons validate our findings and demonstrate that despite Switzerland’s advanced pedestrian infrastructure and healthcare system, elderly pedestrians remain at elevated risk for severe injury and poor outcomes. The consistency of these patterns across high-income countries with varying traffic safety records suggests that age-related physiological factors, rather than system-level factors alone, drive these disparities. This underscores the need for age-specific prevention strategies and clinical management protocols that transcend national boundaries.

### 4.1. General and Study-Informed Recommendations

Our results show that older pedestrians are involved in crosswalk collisions at higher vehicle speeds compared to younger adults. This leads to greater kinetic energy during impact, which is particularly difficult for older individuals to withstand and is associated with more severe trauma [72]. These risks could be eliminated through a combination of measures, such as installing clearly visible speed tables and motion-triggered warning signs, as well as encouraging older pedestrians to wear protective clothing suitable to absorb and distribute kinetic energy during a collision or fall. Additional protective measures—such as rumble strips placed several meters before crosswalks—could further enhance safety by prompting drivers to reduce speed in advance of pedestrian zones. Moreover, priority should be given to enhancing road lighting and promoting the use of night vision assistance systems.

As expected due to the higher incidence of osteoporosis [73], our findings show that fragility-related injuries—such as fractures of the pelvis, upper extremities, and thoracic spine—are significantly more common among older individuals. These injuries are well established to correlate with increased morbidity and mortality [73]. Therefore, in geriatric patients, particularly women, educational initiatives aimed at optimizing bone health—such as smoking cessation programs, regular exercise, and improved physician awareness for the diagnosis and management of osteoporosis—are essential to reduce fracture risk and thereby lower morbidity and mortality in this vulnerable population [3].

Over the past decades, the number of elderly patients presenting to emergency departments has increased significantly, currently accounting for approximately 25% of all visits—a proportion expected to reach 50% by 2030—posing both medical and organizational challenges, including increased demand for physicians [74,75,76,77,78]. Nevertheless, the lack of expertise in the clinical evaluation and therapeutic management of geriatric patients—who frequently present with complex conditions in emergency medicine settings—may significantly worsen prognosis, including increased risks of mortality and disability. Older adults often exhibit atypical clinical presentations, multiple comorbidities, and polypharmacy, all of which raise the risk of diagnostic errors, complicate treatment strategies, and adversely affect clinical outcomes [66,72,79,80]. In fact, emergency department staff frequently report a lack of confidence in managing complex older patients [81], which may be attributed to the limited emphasis on geriatric care in medical and nursing education. To address this gap, future clinicians should aim for a comprehensive understanding of geriatric emergency medicine, including the physiology and pathophysiology of aging, the differentiation between pathological and age-related changes, improvements in education regarding geriatric trauma, the ability to adapt healthcare systems to better meet the needs of older adults, and the capacity to engage with the ethical challenges associated with geriatric care [82,83].

### 4.2. Limitations

Documentation errors cannot be ruled out for any retrospective study, despite our greatest efforts to maintain the best data quality. This single-center design, while allowing for detailed clinical evaluation and consistent documentation, may limit the applicability of our findings to other regions, particularly those with different trauma systems, referral practices, or infrastructure. Additionally, patients with less severe pedestrian trauma may have been treated at peripheral hospitals or private practices, introducing a degree of selection bias that may affect the representativeness of the sample. Although our study spans a ten-year period, the total sample size (n = 124), particularly the elderly subgroup (n = 39), remains relatively small. This limitation is primarily due to the rarity of severe pedestrian injuries specifically occurring at marked crosswalks and requiring admission to a Level I trauma center, as well as the strict inclusion criteria applied. While this reduces statistical power for subgroup analyses and limits generalizability to broader or non-tertiary settings, it ensured a homogeneous cohort and focused analysis. Despite these constraints, the findings are consistent with international data and offer relevant insights for aging populations in high-income countries with comparable infrastructure and urban design [27]. The relatively small sample size also poses theoretical re-identification risks despite our anonymization procedures. However, the risk is mitigated by several factors: (1) the data contains no direct identifiers, (2) quasi-identifiers have been generalized or suppressed where necessary, (3) the study population comes from a large urban catchment area, and (4) data access is restricted to the research team under institutional oversight. Additionally, our study did not include long-term follow-up data, which could provide valuable insights into the persistence of disabilities identified upon presentation at the ED, and could compare short-term and long-term outcomes. Multivariable regression analysis could provide further insights into independent predictors of adverse outcomes, but the limited sample size, especially in the elderly subgroup, precluded robust modeling in the present study. Larger multicenter studies will be necessary to address this gap. Finally, case identification through keywords does not pose a limitation in our setting, as standardized documentation requires the use of these terms. All relevant synonyms, including colloquial expressions, were included, ensuring reliable case ascertainment.

This limitation reflects both the retrospective nature of this study where follow-up was not routinely documented and the lack of access to outpatient and rehabilitation records, as well as the frequent transfer of patients to other facilities or regions following acute care. While we acknowledge this as a limitation, previous research has demonstrated that short-term metrics such as ICU admission [84], ISS [85,86] and LOS [87] are valuable predictors of long-term functional outcomes in trauma and ICU populations. Future research should incorporate these variables prospectively to identify elderly patients at risk of delayed recovery.

## 5. Conclusions

Pedestrian crossing accidents pose a serious and growing public health concern for the elderly, as they are associated with high injury severity, prolonged hospital and ICU stays, and increased mortality rates. Our study highlights the finding that older pedestrians experience disproportionately severe trauma compared to younger individuals, necessitating greater need for hospitalisation and intensive medical care. Given the high risk of life-threatening injuries in this population, EDs must maintain a high level of clinical awareness when treating elderly pedestrian accident victims, thus ensuring timely and specialised care.

Addressing pedestrian safety among the elderly in Switzerland requires a comprehensive approach that integrates behavioural education, stricter enforcement of traffic laws, and strategic improvements in pedestrian infrastructure. By implementing these targeted interventions, such as enhanced traffic regulations, improved crossing safety measures, and public awareness campaigns, the incidence of pedestrian injuries and fatalities can be significantly reduced. Ultimately, these efforts will contribute to better public health outcomes for the aging population, creating a safer and more inclusive urban environment for elderly pedestrians.

## Figures and Tables

**Table 1:** Consultation and patient characteristics by age group comparison. The table presents the absolute number of patients, with percentage shares shown in parentheses relative to the total number of cases studied. * = statistically significant (*p* < 0.05).

Variables	Total	Age < 65	Age ≥ 65	*p* Value
	(n = 124)	(n = 85)	(n = 39)	
Sex				0.53
Female	75 (60.5)	53 (62.4)	22 (56.4)	
Male	49 (39.5)	32 (37.6)	17 (43.6)	
Year of presentation				0.204
2013	13 (10.5)	7 (8.2)	6 (15.4)	
2014	16 (12.9)	12 (14.1)	4 (10.3)	
2015	9 (7.3)	4 (4.7)	5 (12.8)	
2016	14 (11.3)	11 (12.9)	3 (7.7)	
2017	7 (5.6)	7 (8.2)	0 (0.0)	
2018	16 (12.9)	10 (11.8)	6 (15.4)	
2019	10 (8.1)	6 (7.1)	4 (10.3)	
2020	9 (7.3)	9 (10.6)	0 (0.0)	
2021	7 (5.6)	4 (4.7)	3 (7.7)	
2022	13 (10.5)	8 (9.4)	5 (12.8)	
2023	10 (8.1)	7 (8.2)	3 (7.7)	
Month of presentation				0.120
January	19 (15.3)	12 (14.1)	7 (17.9)	
February	10 (8.1)	10 (11.8)	0 (0.0)	
March	8 (6.5)	6 (7.1)	2 (5.1)	
April	4 (3.2)	3 (3.5)	1 (2.6)	
May	3 (2.4)	2 (2.4)	1 (2.6)	
June	4 (3.2)	2 (2.4)	2 (5.1)	
July	7 (5.6)	4 (4.7)	3 (7.7)	
August	10 (8.1)	9 (10.6)	1 (2.6)	
September	12 (9.7)	8 (9.4)	4 (10.3)	
October	14 (11.3)	10 (11.8)	4 (10.3)	
November	15 (12.1)	12 (14.1)	3 (7.7)	
December	18 (14.5)	7 (8.2)	11 (28.2)	
Weekday of presentation				0.245
Monday	25 (20.2)	15 (17.6)	10 (25.6)	
Tuesday	20 (16.1)	13 (15.3)	7 (17.9)	
Wednesday	20 (16.1)	11 (12.9)	9 (23.1)	
Thursday	14 (11.3)	10 (11.8)	4 (10.3)	
Friday	20 (16.1)	17 (20.0)	3 (7.7)	
Saturday	14 (11.3)	9 (10.6)	5 (12.8)	
Sunday	11 (8.9)	10 (11.8)	1 (2.6)	
Time of presentation				0.014
0.01–6.00	6 (4.8)	6 (7.1)	0 (0.0)	
6.01–12.00	32 (25.8)	21 (24.7)	11 (28.2)	
12.01–18.00	40 (32.3)	21 (24.7)	19 (48.7)	
18.01–0.00	46 (37.1)	37 (43.5)	9 (23.1)	
Way of presentation				0.029 *
Self-admission to the hospital	17 (13.7)	15 (17.6)	2 (5.1)	
Ambulance	77 (62.1)	54 (63.5)	23 (59.0)	
Air-Ambulance	13 (10.5)	6 (7.1)	7 (17.9)	
External hospital transfer	12 (9.7)	5 (5.9)	7 (17.9)	
Family doctor or urgent care centre	3 (2.4)	3 (3.5)	0 (0.0)	
Repatriation	2 (1.6)	2 (2.4)	0 (0.0)	
Triage				0.169
Acute life-threating	34 (27.4)	21 (24.7)	13 (33.3)	
High urgency	51 (41.1)	33 (38.8)	18 (46.2)	
Urgency	35 (28.2)	29 (34.1)	6 (15.4)	
Less urgency	4 (3.2)	2 (2.4)	2 (5.1)	
Shock room	68(54.8)	44(51.8)	24(61.5)	0.31
Discharge				0.001 *
Home	65 (52.4)	54 (63.5)	11 (28.2)	
Hospital admission	55 (44.4)	30 (35.3)	25 (64.1)	
Dead in ED	2 (1.6)	1 (1.2)	1 (2.6)	
Transfer to other hospital	2 (1.6)	0 (0.0)	2 (5.1)	
Hospitalisation department				0.020 *
General internal medicine	5 (4.1)	1 (1.2)	4 (10.3)	
Neurology/Neurosurgery	13 (10.6)	7 (8.3)	6 (15.4)	
Intensive care	13 (10.6)	5 (6.0)	8 (20.5)	
Orthopaedics	20 (16.3)	12 (14.3)	8 (20.5)	
Hand surgery	1 (0.8)	1 (1.2)	0 (0.0)	
Thorax surgery	1 (0.8)	1 (1.2)	0 (0.0)	
Visceral surgery	1 (0.8)	1 (1.2)	0 (0.0)	
Stroke	1 (0.8)	1 (1.2)	0 (0.0)	
Rheumatology	1 (0.8)	1 (1.2)	0 (0.0)	

**Table 2:** Description of the accident setting. * = statistically significant (*p* < 0.05).

Variables	Total (n = 124)	Age < 65 (n = 85)	Age ≥ 65 (n = 39)	*p* Value
Mechanism of injury (accident versus)				
Vehicle type group				
One axis	12	8	4	0.883
Two axis	122	77	45	
Vehicle type				
Car	109 (87.9)	75 (88.2)	34 (87.2)	0.607
Bicycle	4 (3.2)	3 (3.5)	1 (2.6)	
E-bicycle	3 (2.4)	1 (1.2)	2 (5.1)	
E-scooter	1 (0.8)	1 (1.2)	0 (0.0)	
Tram	1 (0.8)	1 (1.2)	0 (0.0)	
Motorcycle	4 (3.2)	3 (3.5)	1 (2.6)	
Bus	1 (0.8)	1 (1.2)	0 (0.0)	
Truck	1 (0.8)	0 (0.0)	1 (2.6)	
Accident time				0.187
Day	107 (86.3)	71 (83.5)	36 (92.3)	
Night	17 (13.7)	14 (16.5)	3 (7.7)	
Vehicle speed	30 (15; 40)	30 (15; 40)	37.5 (15; 40)	0.697
Patient hurled	47 (56.6)	26 (47.3)	21 (75.0)	0.016 *
Impact side				
Laterally	112 (91.1)	79 (92.9)	33 (86.8)	0.100
Frontally	9 (7.3)	6 (7.1)	3 (7.9)	
Behind	2 (1.6)	0 (0.0)	2 (5.3)	

**Table 3:** Anatomical injury locations and severity in patients admitted after crossing accidents, grouped by age. * = statistically significant. The table presents the absolute number of patients, with percentages shown in parentheses, relative to the total number of cases studied.

Variables	Total (n = 124)	Age < 65 (n = 85)	Age ≥ 65 (n = 39)	*p* Value
ISS	5 [1; 14]	4 [1; 12]	14 [2; 26]	0.004 *
AIS > 2				
AIS head & neck & cervical spine	23 (18.5)	13 (15.3)	10 (25.6)	0.169
AIS face	3 (2.4)	1 (1.2)	2 (5.1)	0.184
AIS chest & thoracic spine	17 (13.7)	7 (8.2)	10 (25.6)	0.009 *
AIS abdomen & pelvic contents & lumbar spine	3 (2.4)	0 (0.0)	3 (7.7)	0.010 *
AIS extremities & bony pelvis > 2 AIS external	20 (16.1)	8 (9.4)	12 (30.8)	0.003 *
AIS externa l > 2	0 (0.0)	0 (0.0)	0 (0.0)	-

Abbreviations: AIS, abbreviated injury scale; ISS, injury severity score; IQR, interquartile range.

## Data Availability

The data presented in this study are available on request from the corresponding author.

## Abbreviations

The following abbreviations are used in this manuscript:

AIS	Abbreviated Injury Scale
CHF	Swiss Francs
CT	Computed Tomography
ED	Emergency Department
EDH	Epidural Hematoma
EMS	Emergency Medical Services
GCS	Glasgow Coma Scale
HR	Heart Rate
ICB	Intracranial Bleeding
ICH	Intracerebral Hemorrhage
ICU	Intensive Care Unit
IQR	Interquartile Range
ISS	Injury Severity Score
KEK	Kantonale Ethikkommission
LOS	Length of Stay
MRI	Magnetic Resonance Imaging
NTDB	National Trauma Data Bank
SAH	Subarachnoid Hemorrhage
SBP	Systolic Blood Pressure
SD	Standard Deviation
SDH	Subdural Hematoma
TBI	Traumatic Brain Injury

## Supplementary Materials

The following supporting information can be downloaded at https://www.mdpi.com/article/10.3390/ijerph22101556/s1, Table S1: Overview of Key Variables in the Dataset.
