# Supplementary material for: When Pedestrian Crossings Become Danger Zones: Trauma and Mortality Risks in Elderly Pedestrians
Source: Int J Environ Res Public Health. 2025 Oct 13;22(10):1556. doi: 10.3390/ijerph22101556 (PMC12563057; doi:10.3390/ijerph22101556)
Supplement: Supplementary file 1 [file ijerph-22-01556-s001.zip › ijerph-3817018-supplementary.pdf]

Table S1. Overview of Key Variables in the Dataset

| Data Category | Variables | Data Type | Example / Range |
|---|---|---|---|
| Demographics | Age, Sex, Nationality | Numerical / Categorical | 16–95 years; male / female; Swiss |
| Timing of Accident | Year, Month, Day of Week, Time of Day | Categorical | 2019; January; Monday; 18:01–00:00 |
| Accident Characteristics | Vehicle type, Time of day, Vehicle speed, Type of impact | Categorical / Numerical | Car; Daytime; 30 km/h; frontal |
| Injury Characteristics | Trauma type, ISS, AIS, GCS, Loss of consciousness | Numerical / Categorical | Polytrauma; ISS = 14; AIS > 2 (chest); GCS = 15 |
| Clinical Outcomes | Length of stay (LOS), ICU stay, In-hospital mortality, 30-day mortality | Numerical / Categorical | LOS = 9 days; ICU admission; mortality = yes |
